# Patterns of Care and Outcomes Among Women With Locally Advanced Cervical Cancer Treated With Curative Intent at a Tertiary Center in South Africa

**DOI:** 10.1002/cam4.70712

**Published:** 2025-03-13

**Authors:** Juliet Maina, Katie E. Lichter, Elana T. Benishay, Jessica George, Michelle Henry, Nazia Fakie, Surbhi Grover

**Affiliations:** ^1^ Department of Radiation Oncology University of Cape Town, Groote Schuur Hospital Cape Town South Africa; ^2^ Department of Radiation Oncology University of California San Francisco California USA; ^3^ Feinberg School of Medicine Northwestern University Chicago Illinois USA; ^4^ Donald Bren School of Information and Computer Sciences University of California San Francisco California USA; ^5^ Centre for Higher Education University of Cape Town Cape Town South Africa; ^6^ Department of Radiation Oncology University of Cape Town Cape Town South Africa; ^7^ Botswana‐UPenn Partnership University of Pennsylvania Philadelphia Pennsylvania USA

## Abstract

**Objective:**

Cervical cancer is the leading cause of cancer‐related deaths for women in South Africa. The standard of care treatment for locally advanced cervical cancer (LACC) is external beam radiation followed by brachytherapy with concurrent platinum‐based chemotherapy. There exists a paucity of data regarding the treatment regimens received by women with LACC in South Africa. The aim of this study is to assess the patterns of care and survival for patients with LACC treated with curative intent at a tertiary care center in South Africa.

**Materials and Methods:**

This is a retrospective review of cervical cancer patients with histologically confirmed LACC (stage IB2—IVA) who underwent radiation with curative intent at Groote Schuur Hospital in Cape Town, South Africa between July 2013 and July 2018. Overall survival (OS) and disease‐free survival (DFS) were evaluated using the Kaplan–Meier method. Cox proportional hazards modeling analyzed patient and treatment factor associations with survival. Logistic regression modeling was performed to assess factors associated with the receipt of chemotherapy and baseline hemoglobin.

**Results:**

Among 278 patients, 28.4% (*n* = 79) of women had co‐infection with HIV, and 64.8% (*n* = 180) received chemoradiation. Regardless of HIV status, patients who received chemoradiation had improved 2‐year OS (87.4% vs. 52.8%, *p* < 0.001) and DFS (80.2% vs. 58.3%, *p* < 0.001) compared to those receiving radiation alone. Factors associated with improved OS were receipt of chemotherapy (HR 0.32, *p* = 0.005) and higher baseline hemoglobin (HR 0.86, *p* = 0.018). Upon multivariate logistic regression, adjusting for age, stage, and HIV status, patients with stage III/IV disease were less likely to receive chemotherapy (HR 48.17, *p* < 0.001) and were less likely to have hemoglobin ≥ 10 g/dL (HR 0.20, *p* < 0.001).

**Conclusions:**

Addition of chemotherapy to standard radiation improved OS in women with LACC, regardless of HIV status. Our findings add to a body of literature highlighting the importance of providing concurrent chemoradiotherapy to all patients with LACC, including persons living with HIV and those with stage III/IV disease.

## Graphical abstract

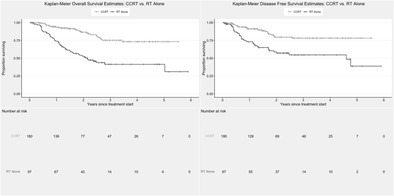

## 1 Introduction

Globally, cervical cancer is the fourth most common cause of cancer‐related death in women. While the incidence and mortality of cervical cancer are decreasing in high‐income countries [1], low‐ and middle‐income countries (LMIC) are still significantly impacted. Nearly 90% of the 311,000 cervical cancer‐related deaths in 2018 occurred in LMICs [2]. In sub‐Saharan Africa (SSA), cervical cancer is the leading cause of cancer‐related death among women [3], and the overall incidence of cervical cancer is increasing [4]. This rise is occurring in tandem with insufficient cervical cancer screening [5, 6, 7, 8] and endemic human immunodeficiency virus (HIV) infection. HIV is associated with increased prevalence and reduced clearance of high‐risk oncogenic human papillomavirus (HPV) strains [9, 10]. This leads to increased prevalence of cervical dysplasia and cancer in those with HIV and HPV co‐infection. In South Africa, a significant number of women are infected with high‐risk HPV and are at risk for progression to cervical cancer [11].

Most women with cervical cancer in South Africa present with locally advanced disease [12], categorized as stage IB2–IVA, according to the International Federation of Gynecology and Obstetrics (FIGO) 2009 staging [13]. The standard of care for locally advanced cervical cancer (LACC) is chemoradiation, established with the publication of five phase III clinical trials that showed a 30%–50% increase in cervical cancer survival rates with chemoradiation compared with radiation alone [14, 15, 16, 17, 18]. Current guidelines recommend external beam radiotherapy followed by intracavitary brachytherapy with concurrent platinum‐based chemotherapy [19].

Even with the current guidelines endorsing concurrent chemoradiotherapy (CCRT) for patients with LACC, many patients across SSA do not receive this care. A prospective study conducted in Zambia compared acute toxicity by HIV status in cervical cancer patients receiving radical CCRT and found that 27% of patients did not receive a second round of cisplatin [20]. In a retrospective analysis from Zimbabwe investigating CCRT initiation practices for women with stage IIIB cervical cancer, 55.7% of patients were prescribed CCRT, but only 37% of patients ultimately received CCRT due to various reasons, including financial constraints [21]. Furthermore, a 2019 survey conducted in 29 centers across 12 sub‐Saharan countries found that despite a majority of women having access to radiotherapy (96%), brachytherapy (85%), and chemotherapy (86%), 33% of centers had to delay or substitute a chemotherapy drug because of inconsistent drug supply [12]. These known limitations have resulted in variability of treatment regimens for women with LACC in sub‐Saharan Africa.

Despite the high incidence and mortality of cervical cancer, there exists a paucity of data describing patterns of treatment and outcomes for cervical cancer patients, with and without HIV, in South Africa. This retrospective review of women with LACC treated with curative intent at a tertiary center in South Africa examines patterns of care, factors related to the receipt of treatment, and associated outcomes.

## 2 Patients and Methods

### 2.1 Study Site and Population

This retrospective review included women with histologically confirmed LACC (stage IB2–IVA) who were treated with curative intent at Groote Schuur Hospital (GSH) in Cape Town, South Africa. Patients were excluded if they underwent primary surgery or were less than 18 years of age.

### 2.2 Data Collection

Patient demographics, including age, clinical history, tumor characteristics (histology and stage), HIV status, and baseline laboratory values (CD4 count, hemoglobin, and creatinine) were collected from medical records.

### 2.3 HIV and Antiretroviral Therapy (ART)

At GSH, patients with LACC are routinely tested for HIV at first presentation and, if positive, are initiated on antiretroviral treatment. Standard first‐line therapy for HIV at the time of the study was co‐formulated Tenofovir, Emtricitabine, and Efavirenz. Data on viral load, compliance, medication changes, and ART toxicities were not available for the study cohort.

### 2.4 Staging and Treatment

Patients were clinically staged by both a gynecological oncologist and a radiation oncologist according to the FIGO 2009 staging system [13]. Baseline imaging, including chest X‐rays and abdominal ultrasounds, was obtained to verify all patients' staging.

Curative treatment during the study period was considered to be receipt of external beam radiotherapy (EBRT), with or without chemotherapy, followed by intracavitary brachytherapy. EBRT was prescribed as 46 Gray (Gy) in 23 fractions to the whole pelvis using 3D conformal radiotherapy techniques or volumetric modulated arc therapy and 6 MV (MV) or 18 MV energies. Concurrent cisplatin (40 mg/m^2^) was given weekly for 4–5 cycles during EBRT unless the estimated glomerular filtration rate was < 60 mL/min/1.73 m^2^, hydronephrosis was present, or poor performance status (defined as ECOG ≥ 3) [22] was present. If cisplatin was contraindicated, carboplatin [area under the curve (AUC) 2] was administered weekly. After completion of EBRT, an additional 14–25 Gy was delivered through high dose‐rate brachytherapy in 2–4 fractions of 5–7 Gy per fraction. At the time of the study, image‐guided brachytherapy was not available. Patients who were not suitable for brachytherapy (i.e., high esthetic risk, elevated body mass index, or a large tumor occluding the cervical os) received an EBRT boost of 18 Gy. The total dose delivered for all patients was calculated as a combined dose of 2 Gy per fraction radiobiologic equivalence (EQD2) [23] as recommended by the American Brachytherapy Society [24]. Upon completion of treatment, women were seen for follow‐up every 3–6 months for the first 1–2 years, and then every 6–12 months for the next 3–4 years if there was no evidence of disease recurrence. During follow‐up, toxicities were assessed clinically and managed appropriately.

### 2.5 Outcomes

The primary outcome for the study was overall survival (OS), defined as the time in years from the start of treatment until death from any cause. Patients lost to follow‐up were censored using the most recent follow‐up visit.

The secondary outcome was disease‐free survival (DFS), defined as the time in years from the start of treatment until disease recurrence, including both local recurrence and distant metastases. Patients lost to follow‐up were censored using the most recent follow‐up visit.

Additional secondary outcomes included receipt of chemotherapy and baseline hemoglobin ≥ 10 g/dL.

### 2.6 Statistical Analysis

Descriptive statistics were used to highlight all collected patient data. OS and DFS were evaluated using the Kaplan–Meier method, with log‐rank tests used to compare survival rates among various subgroups. Factors associated with OS and DFS were analyzed using Cox proportional hazards regression modeling. Logistic regression modeling was performed to assess factors associated with chemotherapy receipt and baseline hemoglobin ≥ 10 g/dL. Statistical analysis was performed using R (RStudio Team, Boston, MA) and statistical significance was considered with a *p*‐value of less than 0.05.

### 2.7 Ethics

Approval was obtained from the University of Cape Town (UCT) Human Ethics Committee.

## 3 Results

### 3.1 Patient Characteristics

A total of 278 women were eligible to participate in this study, of which 28.4% (*n* = 79) were women living with HIV (WLWH) and 71.6% (*n* = 199) were women who were not infected with HIV. Patient demographics, clinical characteristics, and treatment characteristics are summarized in Table 1. Among WLWH, the median CD4 count was 442 cells/μL (IQR; 320–575 cells/μL) and 98.5% (*n* = 66) were receiving ART. Among the cohort (*n* = 278), the median age at diagnosis was 51 years (IQR; 41–60 years). Less than half (34.9%, *n* = 97) of patients reported a smoking history. Most patients had stage II and stage III disease, accounting for 48.6% (*n* = 135) and 45.3% (*n* = 126) of the cohort, respectively. Most women (89.5%, *n* = 246) had squamous cell carcinoma (SCC). The median baseline hemoglobin among all patients was 11.3 g/dL (IQR; 9.7–12.8 g/dL).

**TABLE 1:** Patient demographic, clinical, and treatment characteristics among study participants with LACC treated with curative intent at a tertiary center in South Africa between 2013 and 2018.

Demographics	Total, *N* = 278 (100%)
Age (years), median (IQR)	51 (41–60)
Living with HIV	79 (28.4%)
CD4 (cells/μL)	442 (320–575)
On ART	66 (83.5%)
Smoking, yes	97 (34.9%)
Marital status	
Single	94 (33.8%)
Married	102 (36.7%)
Divorced	32 (11.5%)
Widowed	49 (17.63%)
Tumor characteristics	
Histology	
Squamous	246 (89.5%)
Adenocarcinoma	18 (6.5%)
Others/Unknown	11 (4.0%)
Stage	
I	16 (5.8%)
II	135 (48.6%)
III	126 (45.3%)
IV	1 (0.%)
Baseline investigation, median (IQR)	
Hemoglobin (g/dL)	11.3 (9.7–12.8)
Creatinine (μmol/L)	59 (51–69)
Treatment characteristics	
Concurrent chemotherapy	180 (64.7%)
Number of chemo cycles, median (IQR)	5 (4–5)
Completed brachytherapy	261 (93.9%)
Received external beam radiation	13 (4.7%)
EQD2 (Gy), median (IQR)	74.5 (69–80.9)

*Note:* Numbers are presented as *n* (row %), unless indicated.

Abbreviations: CD4, cluster of differentiation 4 (T cells); EQD2, equivalent dose in 2 Gy fractions; HIV, human immunodeficiency virus; IQR, interquartile range.

### 3.2 Treatment

All patients had LACC and were treated with curative intent. The treatment entailed EBRT with or without concurrent platinum‐based chemotherapy followed by intracavitary brachytherapy. Among all patients, 64.7% (*n* = 180) received chemoradiation and 34.9% (*n* = 97) received radiation alone. Among patients who received chemoradiation (*n* = 180), 94.4% (*n* = 170) received cisplatin and 5.6% (*n* = 10) received carboplatin; the median chemotherapy cycles were 5 cycles (IQR; 4–5 cycles) of concurrent chemotherapy and 5 (2.8%) patients received only 1 chemotherapy cycle. The median EQD2 dose was 74.5 Gy (IQR; 69.0–80.9 Gy). Thirteen patients (4.7%) received external beam radiation instead of intracavitary brachytherapy, while 261 (93.9%) patients received brachytherapy.

### 3.3 Patient Outcomes

The median follow‐up among the cohort was 1.64 years (2.07 years for living patients). Overall, the recurrence rate was 23.0%; recurrence rates were 15.0% and 37.1% for those who received CCRT and radiotherapy alone, respectively. At the end of the study period, 171 (61.5%) patients were alive, 28 (10.1%) were lost to follow‐up, and 79 (28.4%) had died. Causes of death includedlocal recurrence (*n* = 18; 22.8%), metastatic disease (*n* = 27; 34.2%), HIV‐related illness (*n* = 2; 2.5%), unrelated/accidental cause (*n* = 7; 8.9%), and unknown causes (*n* = 25; 31.6%).

### 3.4 OS and DFS

The 2‐year OS and DFS rates among the cohort were 73.3% and 72.3%, respectively. On multivariate Cox regression, adjusting for age, HIV status, stage, hemoglobin, receipt of chemotherapy, and EQD2, factors associated with improved OS were receipt of chemotherapy (hazard ratio [HR] 0.32; 95% CI 0., adjusting for 14–0.69, *p* = 0.004) and higher baseline hemoglobin (HR 0.86; 95% CI 0.76–0.97, *p* = 0.015)—Table 2. Similarly, higher baseline hemoglobin (HR 0.88; 95% CI 0.77–1.00, *p* = 0.047) was associated with improved DFS—Table 2. Our study indicated no difference between HIV‐infected and HIV‐uninfected patients for OS (HR 1.39; 95% CI 0.82–2.33, *p* = 0.221) or DFS (HR 1.13; 95% CI 0.64–1.99, *p* = 0.668).

**TABLE 2:** Predictors of overall survival and disease‐free survival, a univariate and multivariate Cox regression analysis among study participants with LACC treated with curative intent at a tertiary center in South Africa between 2013 and 2018.

Characteristics	All	OS, UVA HR (95% CI)	OS, MVA HR (95% CI)	DFS, UVA HR (95% CI)	DFS, MVA HR (95% CI)^b^
Age (years)					
20–39	51	—	—	—	—
40–59	142	0.77 (0.44–1.35) *p* = 0.359	1.03 (0.56–1.87) *p* = 0.928	0.64 (0.37–1.14) *p* = 0.131	0.84 (0.46–1.52) *p* = 0.560
≥ 60	67	0.79 (0.40–1.52) *p* = 0.476	1.27 (0.59–2.73) *p* = 0.536	0.29 (0.12–0.70) *p* = 0.006^a^	0.48 (0.19‐1.24) *p* = 0.132
HIV status					
HIV negative	199	—	—	—	—
HIV positive	79	1.73 (1.09–2.75) *p* = 0.020^a^	1.39 (0.82–2.33) *p* = 0.221	1.68 (1.00–2.81) *p* = 0.050	1.13 (0.64–1.99) *p* = 0.668
Stage					
I–II	151	—	—	—	—
III–IV	127	3.09 (1.94–4.91) *p* < 0.001^a^	0.96 (0.43–2.15) *p* = 0.927	3.44 (2.04–5.82) *p* < 0.001^a^	2.04 (0.92–4.52) *p* = 0.080
Hemoglobin (g/dL)					
Mean	11.2	0.84 (0.76–0.93) *p* = 0.001^a^	0.86 (0.76‐0.97) *p* = 0.015^a^	0.81 (0.73‐0.91) *p* < 0.001^a^	0.88 (0.77‐1.00) *p* = 0.047^a^
Receipt of chemotherapy					
No	98	—	—	—	—
Yes	180	0.26 (0.17–0.42) *p* < 0.001^a^	0.32 (0.14–0.69) *p* = 0.004^a^	0.34 (0.20–0.55) *p* < 0.001^a^	0.59 (0.28–1.24) *p* = 0.163
EQD2					
Mean	73.9	0.98 (0.96–1.00) *p* = 0.091	0.98 (0.96–1.01) *p* = 0.182	1.01 (0.98–1.03) *p* = 0.721	1.01 (0.98–1.04) *p* = 0.490

Abbreviations: EQD2, equivalent dose in 2 Gy fractions; Hb, hemoglobin; HIV, human immunodeficiency virus; IQR, interquartile range; MVA, multivariate analysis; OS, overall survival; UVA, univariate analysis.

^a^ Statistically significant.

^b^ Multivariate regression adjusting for: age, HIV status, stage, hemoglobin, receipt of chemotherapy, and EQD2.

**FIGURE 1:**
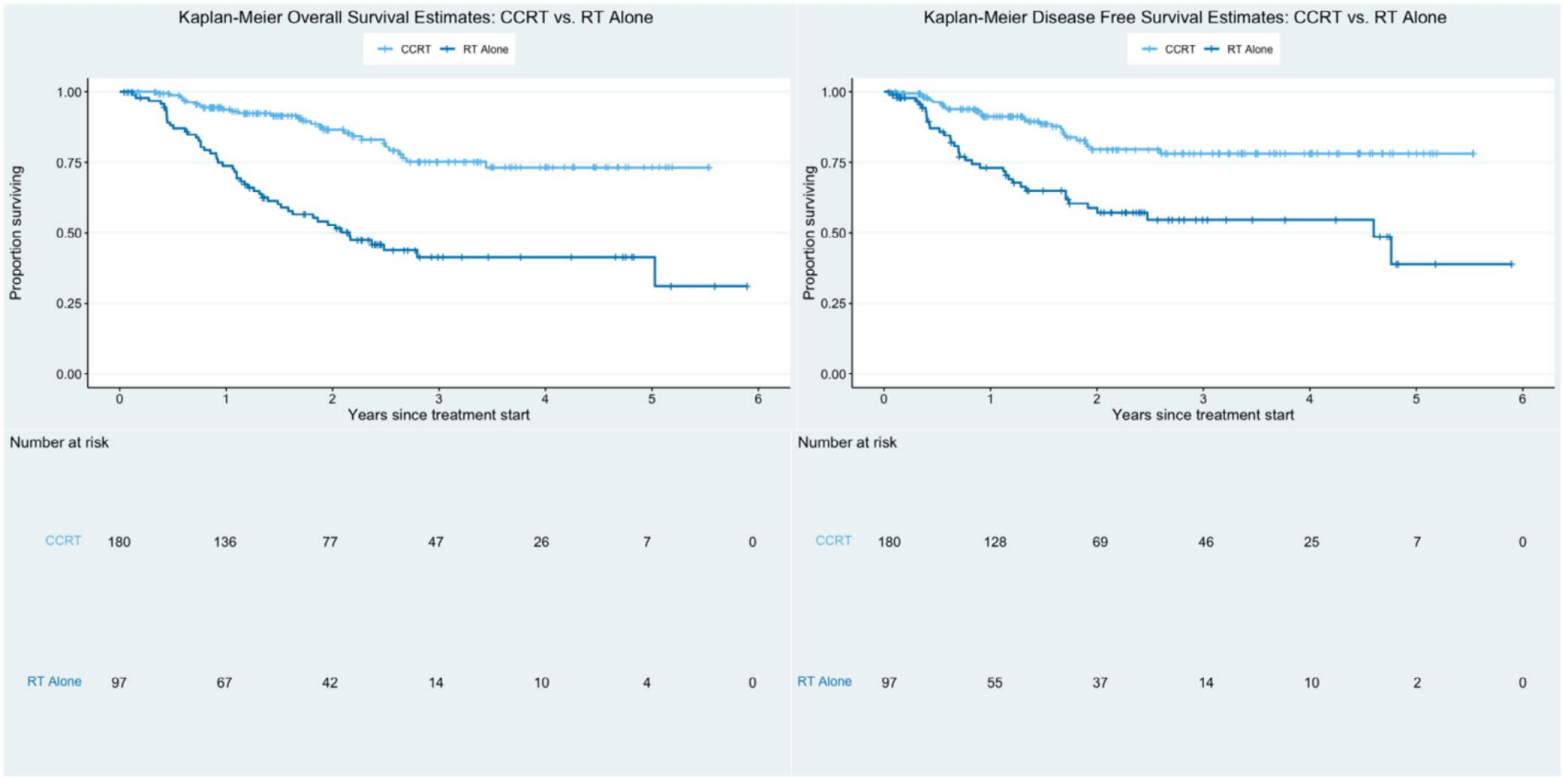
Overall survival (*p* < 0.001) and disease‐free survival (*p* < 0.001) for patients with LACC treated with CCRT versus RT alone in South Africa between 2013 and 2018.

OS significantly differed among patients who received CCRT versus radiotherapy alone (log‐rank, *p* < 0.001); among patients who received CCRT, the 2‐year OS rate was 86.6%, compared to the 2‐year OS rate of 52.8% in those who received radiotherapy alone. DFS also significantly differed (log‐rank, *p* < 0.001); the 2‐year DFS was 79.6% for patients receiving CCRT versus 58.8% for those receiving radiotherapy alone—Figure 1.

### 3.5 Multivariate Analysis

On multivariate logistic regression, adjusting for age, stage, HIV status, and hemoglobin, patients with stages III–IV disease were less likely to receive chemotherapy (odds ratio [OR] 0.02; 95% CI 0.01–0.05, *p* < 0.001) compared to stages I–II—Table 3; When adjusting for age, stage, and HIV status, patients were less likely to have hemoglobin ≥ 10 g/dL (OR 0.20; 95% CI 0.10–0.36, *p* < 0.001) compared to stages I–II—Table 4. Of the patients with stage III–IV disease (*n* = 127), 29.9% (*n* = 38) received chemotherapy, while the remaining 70.1% (*n* = 89) did not.

**TABLE 3:** Predictors for receipt of chemotherapy, a univariate and multivariate logistic regression analysis among study participants with LACC treated with curative intent at a tertiary center in South Africa between 2013 and 2018 (*n* = 278).

Receipt of chemotherapy	Yes	No	UVA OR (95% CI)	MVA OR (95% CI)^b^
Age (years)				
20–39	30 (58.8)	21 (41.2)	—	—
40–59	97 (68.3)	45 (31.7)	1.51 (0.77–2.91) *p* = 0.222	0.94 (0.35–2.45) *p* = 0.894
≥ 60	43 (64.2)	24 (35.8)	1.25 (0.59–2.66) *p* = 0.553	1.21 (0.38–3.86) *p* = 0.752
Stage				
I–II	142 (94.0)	9 (6.0)	—	—
III–IV	38 (29.9)	89 (70.1)	0.03 (0.01–0.06) *p* < 0.001^a^	0.02 (0.01‐0.05) *p* < 0.001^a^
HIV Status				
HIV−	138 (69.3)	61 (30.7)	—	—
HIV+	42 (53.2)	37 (46.8)	0.50 (0.29–0.86) *p* = 0.012^a^	0.43 (0.18–0.99) *p* = 0.051
Hemoglobin (g/dL)				
Mean (SD)	11.4 (2.3)	10.8 (2.1)	1.14 (1.02–1.28) *p* = 0.028^a^	0.85 (0.72–1.01) *p* = 0.063

Abbreviations: EQD2, equivalent dose in 2 Gy fractions; Hb, hemoglobin; HIV, human immunodeficiency virus; IQR, interquartile range; MVA, multivariate analysis; UVA, univariate analysis.

^a^ Statistically significant.

^b^ Multivariate regression adjusting for: age, stage, HIV status, and hemoglobin.

**TABLE 4:** Patient factors associated with hemoglobin ≥ 10 g/dL: a univariate and multivariate logistic regression analysis.

Hemoglobin	< 10	≥ 10	UVA OR (95% CI)	MVA OR (95% CI)^b^
Age				
20–39	22 (44.0)	28 (56.0)		
40–59	37 (26.8)	102 (73.9)	2.23 (1.13–4.38) *p* = 0.020^a^	1.74 (0.81–3.70) *p* = 0.153
≥ 60	11 (17.5)	52 (82.5)	3.71 (1.61–9.02) *p* = 0.003^a^	3.30 (1.28–8.85) *p* = 0.015^a^
Stage				
I–II	19 (13.3)	124 (86.7)		
III–IV	56 (44.4)	70 (55.6)	0.19 (0.10–0.34) *p* < 0.001^a^	0.20 (0.10–0.36) *p* < 0.001^a^
HIV status				
HIV−	46 (23.7)	148 (76.3)	—	—
HIV+	29 (38.7)	46 (61.3)	0.49 (0.28–0.87) *p* = 0.015^a^	0.57 (0.29‐1.12) *p* = 0.099

Abbreviations: HIV, human immunodeficiency virus; Hb, hemoglobin; MVA, multivariate analysis; OR, odds ratio; UVA, univariate analysis.

^a^ Statistically significant.

^b^ Multivariate regression adjusting for: age, stage, and HIV status.

## 4 Discussion

In this retrospective cohort of South African women treated with curative intent for LACC, we found that receipt of chemotherapy and higher baseline hemoglobin were associated with improved OS and DFS, regardless of HIV status and stage of disease. Furthermore, our data revealed that women with stage III/IV disease and WLWH were less likely to receive concurrent chemotherapy. Less than two‐thirds of our cohort received chemotherapy, which is in accordance with previous studies demonstrating varying cervical cancer treatment patterns across sub‐Saharan Africa, particularly in regard to chemotherapy [12, 20, 21]. Our data highlight the lack of uniform care of patients with LACC in South Africa, and the benefit of concurrent chemotherapy in curative treatment.

Our analysis found no difference in survival when providing CCRT to women without HIV infection and WLWH who had been initiated on ART prior to commencing treatment. These findings are consistent with prior research; in a 2020 systematic review, the majority of included studies showed no difference in treatment outcomes for invasive cervical cancer by HIV status [25]. Additionally, a large prospective cohort study examining women with LACC treated with CCRT in Botswana found no difference in OS between WLWH on ART and women without HIV [26]. Our data emphasize the importance of CCRT for the treatment of LACC with curative intent in a resource‐limited setting, regardless of HIV status.

Similar to the lower rates of chemotherapy in WLWH, we found that patients with stage III‐IV cervical cancer were less likely to receive chemotherapy. There has been conflicting historic data regarding the utility of chemotherapy in women with LACC. Previous studies have found significantly improved overall survival in women with LACC receiving chemoradiation [14, 15, 17]. Similarly, meta‐analyses have found that CCRT improves survival among all cervical cancer patients, though the survival benefit of dual therapy decreases with increasing stage [27, 28, 29]. However, other data have shown that CCRT provides no benefit in advanced disease stages. Two randomized phase III trials demonstrated no significant survival benefit for CCRT over radiotherapy alone in patients with stage IB‐IVA disease [30, 31]. Nonetheless, recent data including a randomized control trial (RCT) from India and a cohort study from Botswana (which included patients with HIV) suggest a benefit with CCRT over RT (Radiotherapy) in stage IIIB [32, 33]. While the benefit for CCRT for stage IIIB patients is clear from the more modern studies, in a resource‐limited environment, concern for decreased utility based on historic data or lack of utility with concurrent chemotherapy for LACC may deter its use.

Another important variable associated with survival is baseline hemoglobin. Our data demonstrated that higher baseline hemoglobin in patients with LACC is associated with improved OS and 2‐year DFS. This aligns with outcomes in other studies evaluating radiotherapy for cervical cancer; higher hemoglobin has been associated with improved survival [34, 35, 36] while lower hemoglobin levels are shown to portend poorer prognosis [37, 38, 39, 40, 41, 42, 43, 44]. It has been suggested that hemoglobin levels influence tumor oxygenation, therefore impacting radioresistance [45]. Blood transfusions may be considered in anemic patients to combat this theoretical hypoxia‐induced radioresistance and improve overall outcomes [35, 46, 47], though this remains controversial [48].

Despite guidelines recommending transfusion of anemic patients treated in our cohort, many patients still had low hemoglobin. Persistent anemia may be due to inadequate transfusion because of limited resources to facilitate this. Another consideration is that severe illness prevented normalization of hemoglobin even after receiving transfusion. In our study, patients with stages III–IV disease were less likely to have baseline hemoglobin ≥ 10 g/dL. It is possible that low baseline hemoglobin levels, signifying an overall diminished state of health, contributed to lower receipt of chemotherapy in patients with more advanced disease. This may reflect a physician bias to omit chemotherapy due to concern for patients' abilities to tolerate an aggressive treatment regimen.

This study is not without limitations. Using a retrospective design, we cannot ascertain why a greater proportion of patients with stage III–IV disease did not receive chemotherapy. However, in this study, we did not evaluate the number of patients with stage IIIB, who may have had hydronephrosis or renal impairment, as this may have been a contributing factor for not receiving chemotherapy. Due to resource limitations, most patients who had hydronephrosis had no intervention, such as stenting or nephrostomy, done in order to improve renal function and further allow for chemotherapy to be administered. Considering limitations in operating theater availability, we did not want decompression of hydronephrosis to delay definitive treatment. This may further have led to provider hesitation or bias. The study data do not include clinical decision‐making regarding the type of radiotherapy received and thus cannot account for provider bias implemented when prescribing brachytherapy versus a boost. Further, we did not include toxicity data, which is an important predictor of survival. Another important consideration is the outdated staging, which was done according to 2009 FIGO criteria. The updated 2018 FIGO staging criteria include surgical pathologic and imaging findings, resulting in upward stage migration for many patients, often due to nodal and distant metastasis [49]. Newer staging may have resulted in different outcomes by disease stage. For WLWH, one of the limitations of our study was that antiretroviral medication toxicities and HIV‐related morbidity were not documented. Despite these limitations, this study remains the first of its kind, both characterizing the cervical cancer patients in South Africa and detailing the treatment regimens received.

With increasing rates of cervical cancer in South Africa, it is imperative that we improve patients' access to standard‐ofof‐care treatments. The results of our study reinforce the body of evidence that concurrent chemoradiotherapy improves outcomes for LACC, regardless of disease stage or HIV status. Future research should focus on understanding systemic and provider barriers and further factors that influence the distribution of appropriate care. Understanding current treatment selection and associated outcomes can guide efforts to standardize CCRT for women undergoing curative treatment for cervical cancer in South Africa.

## Author Contributions

**Juliet Maina:** conceptualization (lead), investigation (lead), methodology (lead), writing – original draft (lead). **Katie E. Lichter:** conceptualization (equal), formal analysis (lead), methodology (equal), resources (equal), writing – original draft (equal). **Elana T. Benishay:** methodology (supporting), writing – original draft (supporting). **Jessica George:** conceptualization (supporting), formal analysis (supporting), writing – original draft (supporting). **Michelle Henry:** formal analysis (supporting). **Nazia Fakie:** conceptualization (lead), investigation (supporting), supervision (lead), writing – original draft (supporting). **Surbhi Grover:** conceptualization (lead), formal analysis (lead), supervision (lead).

## Disclosure

The authors confirm that the work submitted is original and does not transgress the plagiarism policy of the journal. No data, text, or theories by others are presented as if they were the author's own. Proper acknowledgments of other's work have been given (this includes material that is closely copied, summarized and/or paraphrased); quotation marks are used for verbatim copying of material. Permissions have been secured for material that is copyrighted.

## Consent

University of Cape Town, Faculty of Health Sciences—Human Research Ethics Committee. HREC REF: 717/2019. This was a retrospective project, so ethics approval was obtained, but no consent was obtained from the patients. The study involves advanced cervical cancer, and unfortunately, many of the patients had died when the analysis was done. Hence, it was not feasible to obtain consent retrospectively.

## Conflicts of Interest

The authors declare no conflicts of interest.

## Data Availability

The authors confirm that the data supporting the findings of this study are within the article. Raw data that supports the findings of this study are available from the corresponding author, upon request.
